# Sequential Transfer Learning for Multi-Domain Breast Image Segmentation Using a Transformer-Enhanced Hybrid U-Net

**DOI:** 10.3390/bioengineering13050570

**Published:** 2026-05-18

**Authors:** Shagufta Manzoor, Javaria Amin, Amad Zafar

**Affiliations:** 1 Department of Computer Science, University of Wah, Wah Cantt 47040, Pakistan; 2 Department of Computer Science, Rawalpindi Women University, Rawalpindi 43600, Pakistan; 3 Department of Artificial Intelligence and Robotics, Sejong University, Seoul 05006, Republic of Korea

**Keywords:** incremental learning, segmentation, imaging, residual, dice

## Abstract

Worldwide, breast cancer is the leading cause of death in women. This emphasizes the significance of an accurate breast cancer detection system. This study presents a unified framework for segmentation of breast cancer using multimodal imaging, such as histopathology, MRI, mammogram, and ultrasound. This framework integrates the CNN with Transformer modules and has three core technical innovations. First, features are extracted using an encoder–decoder design. The encoder has Residual Blocks with a base channel of 32, following feature extraction, which are progressively mapped and downsampled into four stages (32 → 64 → 128 → 256) of channels. The spatial channel is reduced using MaxPool2d operations from 256 × 256 to 128 × 128, 64 × 64, 32 × 32, and 16 × 16. After further convolutional refinement, a Transformer encoder is used on the 16 × 16 feature maps in the bottleneck. The Transformer comprises four encoders with multi-head self-attention (eight heads) and a 4.0 MLP ratio, enabling the model to capture local and global contextual dependencies at the lowest resolution. The proposed framework is trained with a learning rate of 1 × 10^−4^, up to 50 epochs with early stopping (patience = 12), using a combined Dice and binary cross-entropy loss that balances pixel-wise accuracy and overlap-based learning. Gradient clipping with a maximum norm of 5.0 is used to ensure training stability; ReduceLROnPlateau (factor = 0.5, patience = 5) is used to dynamically adjust the learning rate; and early stopping is used to prevent overfitting. To improve generalization and enhance robustness to data variability, data augmentation techniques such as random horizontal and vertical flips, intensity variations, and small rotations (±15°) are applied. Incremental learning was implemented in this study as a warm-start fine-tuning strategy, where the model was initialized based on learned weights from a previously trained model instead of training from scratch. This is done by loading saved checkpoints of the best-performing model and continuing training on a new dataset. The performance of the proposed framework is evaluated on four publicly available datasets and one local dataset, such as BUS-UCLM, BUSI, BreastDM, TNBC NucleiSegmentation, and BCSD-2024. The impressive results are achieved with Dice scores of 0.974 on ULCM, 0.975 on BUSI, 0.971 on BreastDM, 0.904 on TNBC nuclei segmentation, and 0.982 on BCSD-2024. The proposed model consistently performed better than classical U-Net models.

## 1. Introduction

Breast cancer is a critical health issue in the world. Breast cancer is one of the types of cancer among women. Mostly women die due to this disease; timely and correct diagnosis of breast cancer can save lives and improve the results of the cure [1]. Histopathological analysis plays a golden role in diagnosis. It analyses biopsy samples using a microscope. However, manual testing is slow and subjective. Different pathologists can give different opinions because there is a variation in judgment among observers [2]. Mammography is primarily used for initial diagnosis. It can detect suspicious sores before they become prominent and produce symptoms. But mammogram interpretation is challenging. Very small abnormal tissue, such as tiny calcium spots, tissue distortion, or spiky masses, is difficult to detect against normal tissue [3]. There are often differences among radiologists who read a given set of mammograms because they view them differently. This can create false-positive results and add unnecessary biopsies to the patient population, and false-negative results are caused by creating delays in the patients’ diagnoses and subsequent treatment. Ultrasound is commonly used, particularly with patients with dense breast tissue, to detect smaller lesions than mammograms can identify; however, ultrasound is often noisier in speckle mode, lacks contrast, and has difficulty defining lesion boundaries. The operator’s skill level has a major impact on the accuracy and reliability of ultrasound interpretation. These inconveniences and difficulties make accurate diagnosis difficult, thereby delaying patients’ access to proper treatment as soon as possible [4,5].

Recently, deep learning (DL) methods have been employed for breast cancer detection [6]. These models have been trained on lesion features to discriminate between benign and malignant breast tissue. The limitation of CNNs is that they typically cannot model long-range dependencies because of the inherent locality of convolutional operations; therefore, they focus on parts of the mammogram at any given time while ignoring the rest. Also, due to the computed convolutional operations, the process will be computationally intensive. Common diagnostic tests include mammograms, biopsies, and histopathology. Although these methods provide effective results, they still require significant manual effort and are prone to human error [7]. With the exponential growth of DL, automated imaging methods have been developed to revolutionize breast cancer detection and diagnosis [8].

Therefore, a Multimodal Transformer (MMT) is necessary due to the constraints of convolutional neural networks (CNNs) on local extraction mechanisms for representing subtle malignant alterations, as they are unable to model long-range dependencies and global context [9,10,11].

Accurate detection is still a challenge because ultrasound and histopathology images can vary in quality and magnification. Hybrid models that incorporate CNNs, Transformers, and other architectures show promise but require continued investigation [12]. Compared with existing studies, this research proposes a hybrid framework that provides significant improvements over the classical U-Net through the following key technical innovations.

- This research introduces a new expert-annotated clinical mammography dataset consisting of right and left breast slices from 3042 mammography scans of 10 patients. For each image, manual annotations and corresponding expert-verified ground-truth masks were generated to support breast abnormality detection and segmentation.
- The features are extracted using an encoder–decoder design. The encoder has Residual Blocks with a base channel of 32, following feature extraction, which are progressively mapped and downsampled into four stages (32 → 64 → 128 → 256) of channels. This spatial channel is reduced using MaxPool2d operations from 256 × 256 to 128 × 128, 64 × 64, 32 × 32, and 16 × 16. After further convolutional refinement, a Transformer encoder is applied to the 16 × 16 feature maps in the bottleneck. The Transformer comprises four encoders with multi-head self-attention (eight heads) and a 4.0 MLP ratio, enabling the model to capture local and global contextual dependencies at the lowest resolution.
- The proposed framework is trained with a learning rate of 1 × 10^−4^, 50 epochs, and a combined Dice and binary cross-entropy loss that balances pixel-wise accuracy and overlap-based learning. Gradient clipping with a maximum norm of 5.0 is used to guarantee training stability; ReduceLROnPlateau (factor = 0.5, patience = 5) is used to dynamically control the learning rate; and early stopping criteria is used to avoid overfitting. Data augmentation is also applied with random vertical and horizontal flips, intensity variations, and small rotations (±15°). Incremental learning was implemented in this study as a warm-start fine-tuning strategy, where the model was initialized based on learned weights from a previously trained model instead of training from scratch. This is done by loading saved checkpoints of the best-performing model and continuing training on a new dataset.

The article is organized as follows: related work is discussed in Section 2, the proposed method is explained in Section 3, results and discussion are presented in Section 4 and Section 5, and the conclusion is presented in Section 6.

## 2. Literature Review

A Wavelet-Transformer Hybrid Network (WTHN) was proposed for the processing of blurred and noisy ultrasound images. Wavelet decomposition enabled the extraction of high-frequency texture details and low-frequency shape details. This Transformer model extracted global contextual dependencies using multi-head self-attention. The segmentation map of a high-resolution build using a decoder. The model was analyzed based on the BUSI dataset using accuracy, Dice score, and F1 score. The module analysis has verified the importance of both wavelet and Transformer components [13,14]. M2G-HBoost was introduced to handle noisy, imbalanced data well. It combined Graph Convolutional Networks, Genetic U-Net, and a boosting classifier into an integrated framework. The model achieved the best Dice and IoU scores on the BUSI dataset and classified into normal, benign, and malignant cases [15]. The MammoFormer framework is designed to integrate a Transformer and a CNN, with feature enhancements such as HOG and histogram equalization, to improve interpretability and sensitivity [16].

Multi-Head CNNs were proposed for breast density segmentation and BI-RADS prediction, i.e., the MH-CNN model simultaneously predicted breast density and classified BI-RADS from breast mammograms [17].

Some works have also studied Transformer-based models for full mammogram image detection by incorporating segmentation or mask generation into the pipeline to guide the model to specific tissue regions [18]. Open challenges still lie ahead in mammographic applications: many methods focus on classifying the entire image rather than on accurate segmentation; boundaries of lesions, microcalcifications, and distortions remain difficult to detect in dense breast tissue; available datasets tend to be small or non-diverse in terms of the institutions of origin. The BreastXploreAI model is proposed, featuring a Transformer-based deep learning architecture that synergistically combines Sequential [temporal] encoding Transformers and CNNs for spatial feature extraction. The dual-head classifier was used for cancer subtype and disease stage predictions; both uncorrected and corrected results using the proposed method were presented. The proposed model was a flexible, scalable approach to breast cancer detection, leveraging explainability modules to enhance understanding and generating synthetic temporal sequences to aid learning [19]. To address class imbalance in the BIRADS dataset, this work proposes a multi-stage approach that leverages a pre-trained ResNet50 model to extract features from mammogram images and uses SMOTE for over-sampling the minority class. Keras Tuner was used to optimize the model, and five-fold cross-validation was conducted for training. The detection accuracy of MammoViTis 97%. A 4% detection accuracy in BIRADS categories was confirmed using a series of assessment indicators, including a confusion matrix [20].

A novel real-time mammogram detection approach is introduced that can differentiate between benign and malignant cases. It exploited selective image preprocessing to enhance quality, applied a convolutional neural network (CNN)-based transfer learning (TL) for comprehensive feature analysis, and a vision transformer (ViT) for contextual analysis. The performance of the proposed approach for detection is verified by 98.26% accuracy, an F1-score of 92.58%, 93.25% precision, 95.45% recall, and an area under the receiver operating characteristic (ROC) curve (AUC) of 0.96 [21].

This work aimed to explore the potential of combining convolutional neural networks (CNNs) and vision transformers (ViTs) to enhance radiologists’ manual mammogram interpretation performance. In this work, we experimented with pre-trained models (DenseNet, Inception, SE ResNet, and Xception) and a CNN + ViT model on CLAHE-enhanced mammogram images from Kaggle. XceptionNet was outperformed by CNN + ViT both in terms of precision and accuracy. While XceptionNet achieved 100% accuracy, it still showed signs of overfitting. The CNN + ViT model reached an accuracy of 90.1% [22]. About 2.3 million new cases of breast cancer are recorded every year, making it the most common disease among women. Early detection is crucial to reducing mortality. This paper proposed a hybrid approach based on a Bayesian-Optimized Fast Learning Network (FLN) and DenseNet201-based transfer learning for breast cancer detection using ultrasound images. The model’s accuracy, precision, recall, and F1 score were 96.79%, 94.71%, 93.48%, and 96.81%, respectively. The AUCs for benign, malignant, and normal classes demonstrated good performances, 0.96, 0.95, and 0.98, respectively, while misclassifications were minimized. This new approach outperformed existing models and can serve as a breast cancer diagnostic tool [23]. BUS segmentation has matured since the introduction of deep learning, most recently with attention and hybrid architectures. Context learning, such as SwinEff-AttentionNet, a hybrid model integrating Swin Transformers, EfficientNet blocks, and Efficient Local Self-Attention, achieved state-of-the-art segmentation results, improving lesion localization and diagnostic accuracy [24]. The DMFormer model has been demonstrated to significantly enhance breast cancer detection in ultrasound images, surpassing conventional CNNs and state-of-the-art Transformer-based models [25].

Another work presented a hybrid architecture that combines VGG16 with vision transformers (ViTs) for breast cancer detection, exploiting the multi-head attention mechanism to learn global context more efficiently than traditional CNNs. The binary benign-malignant detection accuracy of the method was 93% in the BreakHis dataset and 95.6% in the BRACS dataset [26]. Hybrid architectures and attention mechanisms have also been used to extract both low-level local details from CNNs and high-level global contexts from Transformer/attention mechanisms. Highlights include hybrid attention networks (e.g., BUS) that integrate global spatial/pair tokens or positional encoding with standard CNNs to enhance boundary definition and mitigate noise sensitivity [9]. In this work, we proposed addressing the challenges in breast cancer detection by combining vision transformers and convolutional neural networks (ViT-CNNs). We introduced an unsupervised stain normalization based on stain identification. This network consisted of two variable parts: a variable attention module to model fine-grained structures and a variable convolution module to capture multi-scale features. Comprehensive evaluations reveal that the proposed method outperforms other existing recent approaches by achieving binary detection accuracy, recall, precision, and F1-score of 98.76%, 98.56%, 97.63%, and 98.49%, respectively as well as multi-class detection accuracy, recall, precision, and F1-score of 96.58%, 96.69%, 96.51, and 96.64% by using publicly accessible breast cancer histopathology images [27]. Yet there are still gaps: there is little work on the unification of all three imaging modalities (mammography, BUS, histology) in a single framework; segmentation boundaries tend to be less accurate; computational and memory costs are prohibitively high for Transformers; and interpretability [28].

## 3. Proposed Methodology

**Figure 1:**
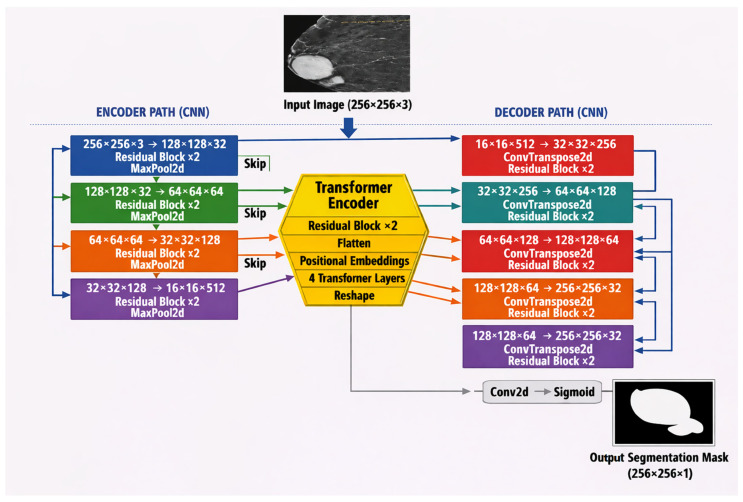
Proposed CNN–Transformer segmentation framework showing encoder-based feature extraction, Transformer-based global feature learning, skip connections, decoder-based feature reconstruction, and final tumor mask generation.

The proposed model workflow, shown in Figure 1, consists of four stages based on Conv2D, BatchNorm, and ReLU, with progressive downsampling. The Transformer block at stage 4 captures global features via patch embedding and multi-head attention. The features are processed in a bottleneck before the decoder performs four stages of upsampling using transposed convolutions with skip connections from the corresponding encoder layers. The output layer generates the final mask using a 1 × 1 convolution and a sigmoid activation. The model is trained using a combined binary cross entropy (BCE) with dice loss to balance boundary and region optimization. The ablation agent manages the architecture variant, such as CNN/Transformer/Loss selection, while the incremental module handles resume-fine-tuning across datasets. The proposed system automatically generates comprehensive artifacts, including training logs, visualization plots, and checkpoints, to complete experiment tracking.

### 3.1. Hybrid U-Net with Transformer Bottleneck Framework

The unified model is based on a hybrid CNN–Transformer–U-Net architecture that integrates local and global feature extraction for breast cancer segmentation. Through embedding a Transformer encoder at the deepest level of the residual U-Net, the model captures the long-range dependencies vital for understanding the morphology of the lesion while preserving the fine details based on skip connections. This architecture describes how synergy between CNNs and Transformers might effectively address the challenges of medical image segmentation, where local and global shape constraints are crucial for better delineation of pathological structures.

#### 3.1.1. CNN Encoder

**Figure 2:**
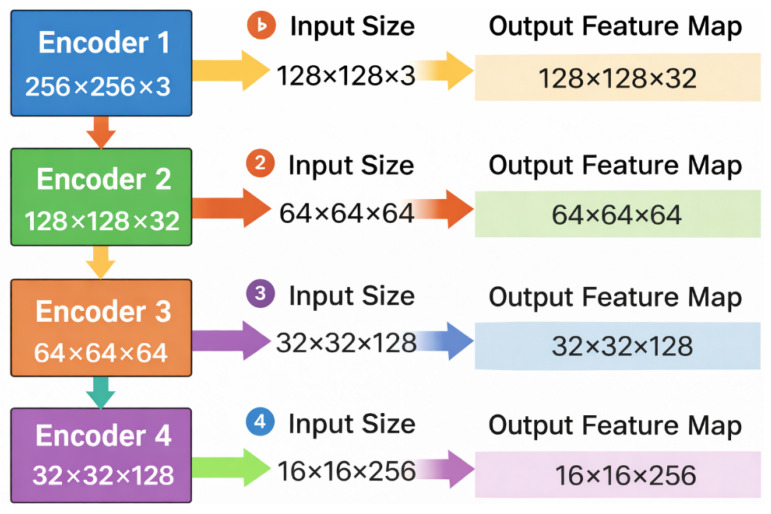
Encoder blocks with multi-scale feature extraction. The input image is progressively downsampled across four encoder stages, reducing spatial resolution while increasing channel depth, generating hierarchical feature maps used for Transformer-based bottleneck processing and decoder skip connections.

The encoder is implemented as a sequence of four Encoder Blocks, each consisting of two Residual Blocks followed by a MaxPool2d layer to reduce the spatial resolution, as shown in Figure 2. The base number of feature channels starts at 32 and doubles after each stage: 32 × 64 × 128 × 256.

Each Residual Block includes two convolutional layers with batch normalization and ReLU activation. The 2D convolution operation at a specific location (i, j) in the output feature map for output channel c is defined by Equation (1):

(1) $$y_{c,i,j}=\sum_{u=0}^{k-1}\sum_{v=0}^{k-1}\sum_{m=0}^{C_{in}-1}x_{m,i+u,j+v}.w_{c,m,u,v}+b_{c}$$

where k is the kernel size, Cin is the number of input channels, and w are the learnable weights. The number of parameters in a convolutional layer is given as Equation (2):

(2) $$Params=C_{out}\times C_{in}\times k^{2}+C_{out}(if\;bias=True)$$

In this implementation, bias is often disabled, and BatchNorm provides affine transformations. The Residual Block applies two such convolutions and includes a skip connection, optionally using a 1 × 1 convolution to match the input and output channel dimensions: Y = ReLU (BN (Conv2d (X; W1))), Z = BN (Conv2d (Y; W2)), S = X if Cin = Cout, else S = BN (${Conv2d}_{1\times 1}$(X; Ws)) otherwise Output = ReLU (Z + S).

#### 3.1.2. Bottleneck (CNN and Transformer Integration)

**Figure 3:**
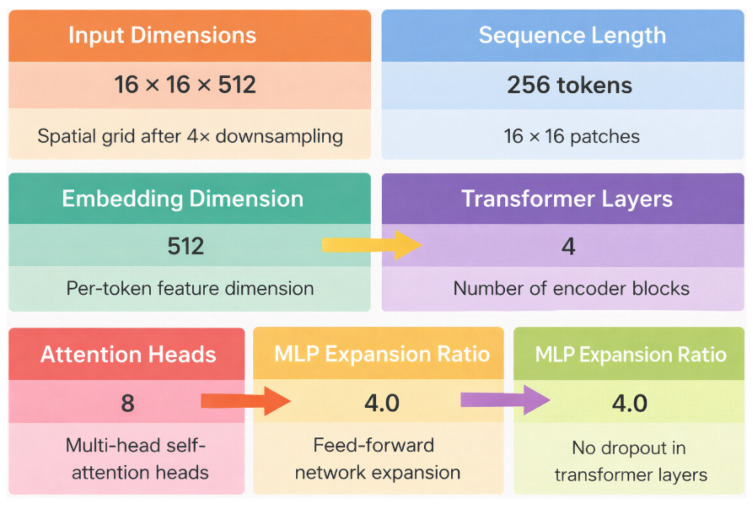
Bottleneck CNN integrated with Transformer layers for global feature modeling. The reduced feature map (16 × 16 × 512) is converted into 256 tokens with 512-dimensional embeddings, then processed by 4 Transformer encoder blocks with 8 attention heads and an MLP expansion ratio of 4.0 to capture long-range dependencies and enhance segmentation performance.

The bottleneck stage combines CNN and Transformer layers to capture both local and global features. After the final encoder block, the feature map $X\in R^{B\times C\times 16\times 16}$ is flattened into tokens $T\in R^{B\times N\times C}$, where N = H × W = 256. Learnable positional embeddings $P\in R^{1\times N\times C}$ are added to these tokens: T′ = T + P, as shown in Figure 3.

These tokens are then passed through L layers of Transformer blocks, where each block contains an MHSA layer and an FFN layer, both with residual connections. The attention mechanism calculates queries, keys, and values as Q = X$W_{Q}$, K = X$W_{K}$, V = X$W_{V}$, and the attention scores are computed using scaled dot-product attention: $Attention\left(Q,K,V\right)=softmax\left(\frac{QK^{T}}{\sqrt{d_{k}}}\right)V$.

In multi-head attention, outputs of individual heads are concatenated and passed through a linear projection: MHSA(X) = Concat (${head}_{1}$...${head}_{H}$)$W_{o}$. Each Transformer block updates tokens using z′ = z + MHSA (LN (z)), z″ = z′ + FFN (LN (z′)). This enables the network to capture long-range dependencies across spatial positions and complements the CNN’s local feature extraction.

#### 3.1.3. Decoder

**Figure 4:**
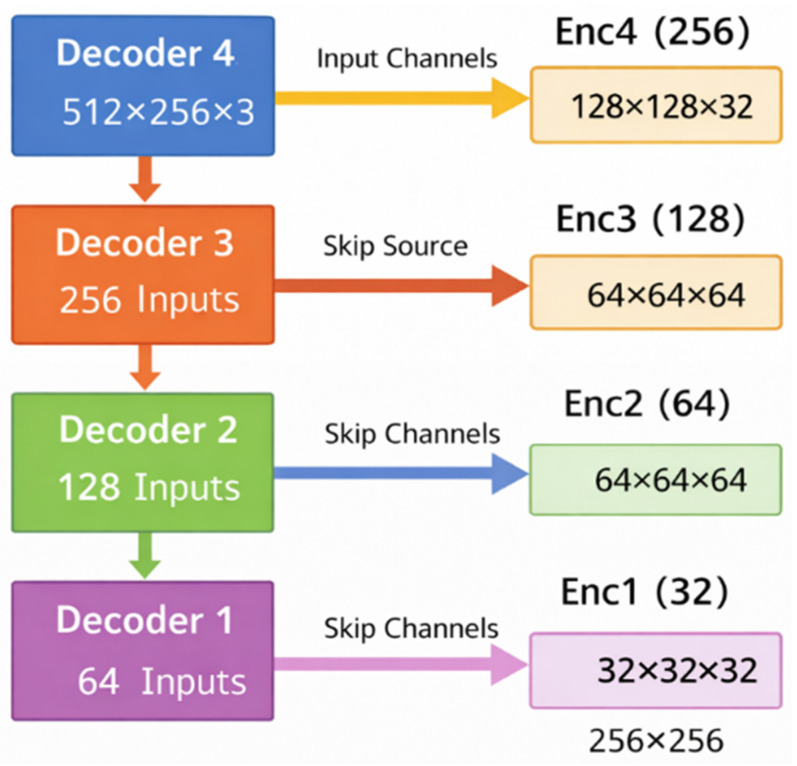
Proposed decoder architecture illustrating multi-scale skip connections between encoder and decoder blocks. Decoder stages progressively upsample feature representations while integrating corresponding encoder features (Enc1–Enc4) via skip connections, preserving fine-grained spatial details and contextual information for accurate segmentation.

The decoder upsamples the segmentation map and refines features by using skip connections from the encoder to the decoder, as shown in Figure 4. Each Decoder Block performs learned upsampling via ConvTranspose2d, concatenates the upsample features with the encoder features at the corresponding resolution, and passes the result through Residual Blocks.

For instance, in Dec4, a 512 × 256 transposed convolution is followed by concatenation with Enc4, then two Residual Blocks process the combined features. This pattern continues [Dec3: 256 × 128, Dec2: 128 × 64, Dec1: 64 × 32]. In case of mismatched tensor shapes (due to rounding or odd dimensions), F. interpolate aligns them before concatenation.

#### 3.1.4. Output Layer and Prediction

The final decoder output has shape (B, 32, 256, 256), which is passed through a 1 × 1 Conv2d to produce a single-channel output for binary segmentation. The resulting logits $z\left(x,y\right)$ are transformed into probabilities using the sigmoid function (Equation (3)):

(3) $$P\left(x,y\right)=\frac{1}{1+exp(-z\left(x,y\right))}$$

During inference, predictions are binarized by thresholding the probability map at 0.5. During training, the model uses BCE with sigmoid for probability estimation.

#### 3.1.5. Loss Functions (Detailed Formulas and Gradients Intuition)

To improve segmentation quality, especially in class-imbalanced datasets, the model combines BCE with Dice loss as Equation (4):

(4) $${ℶ}_{BCE}=\frac{-1}{N}\sum_{i=1}^{N}[y_{i}\log\left(p_{i}\right)+\left(1-y_{i}\right)\log\left(1-p_{i}\right)$$

The differentiable Dice coefficient is defined as Equation (5):

(5) $$Dice=\frac{2\sum y_{i}p_{i}+\epsilon }{\sum p_{i}+\sum y_{i}+\epsilon }$$

where ($p_{i}$) are predicted probabilities, ($y_{i}$) are ground-truth labels, and ϵ is a small constant for numerical stability. The Dice loss is ${ℶ}_{Dice}$ = 1 − Dice. The total loss function is ${ℶ}_{total}$ = ${ℶ}_{BCE}$ + ${ℶ}_{Dice}$. This encourages both per-pixel accuracy and high overlap between the predicted and ground-truth regions.

In Table 1, the architecture of the hybrid U-Net consists of an encoder–decoder module that progressively processes 256 × 256 images through four hierarchical encoder blocks, each employing residual connections and max pooling to systematically reduce spatial dimensions and expand the feature channel from 32 to 256. The core model innovation lies in the bottleneck Transformer, where the 512-channel feature map is processed at 16 × 16 resolution across four Transformer layers with 8 attention heads and a 4× MLP ratio, enabling global context modeling with 33.6 million parameters. The decoder path reconstructs spatial resolution using four blocks that integrate skip connections with residual operations, gradually reducing the feature dimension from 256 × 32 × 32 to 32 × 256 × 256. The architecture outputs a 1 × 1 convolutional layer that generates a binary segmentation mask using the sigmoid activation function. The model has approximately 52.9 million parameters while maintaining precise spatial localization through a CNN–Transformer design. The model parameters and their corresponding memory are given in Table 2.

The hybrid U-Net model is a larger, more complex architecture compared to the simple U-Net model. The hybrid U-Net model’s capacity increases significantly by incorporating a Transformer bottleneck, which accounts for the majority (~63%) of the model’s parameters. This increment enables global context modeling but comes at the cost of higher memory and computational requirements than the lightweight CNN-based U-Net.

### 3.2. Modular Automation

The training pipeline is organized into modular components that handle configuration management, transfer learning, and evaluation. These components are not independent learning agents; rather, they are rule-based controllers that structure and automate the training process. The configuration-driven design is used to define and execute different variants of experiments using a structured list of settings, where key components such as the loss function, hybrid architecture, and reproducible comparisons are specified. During training, validation metrics, such as validation Dice scores, are measured after each epoch and used to guide optimization. The validation data is fed to the scheduler of ReduceLROnPlateau, which dynamically adjusts the learning rate when performance plateaus. It is also used for early stopping, which terminates training if no improvement is observed for a predefined number of epochs. The best model is selected based on validation performance, confirming effective checkpointing and monitoring training. For incremental learning, the framework assumes a warm-start fine-tuning strategy, where previously learned weights are loaded using model.load_state_dict(prev_state, strict = False) to initialize training on a new dataset. This enables the reuse of learned representations, which supports transfer learning across datasets, particularly helpful in medical imaging scenarios with limited labeled data. The approach does not include an explicit regularization mechanism to prevent catastrophic forgetting and therefore relies on sequential fine-tuning rather than constraint-based continual learning methods.

### 3.3. Incremental Weight Transfer

Incremental learning was implemented in this study as a warm-start fine-tuning strategy, where the model was initialized based on learned weights from a previously trained model instead of training from scratch. This is done by loading saved checkpoints of the best-performing model and continuing training on a new dataset. In practice, weights are transferred using a loading mechanism with strict = false, which permits partial compatibility and flexibility in mapping parameters between tasks, unlike regularization-based continual learning methods such as Elastic Weight Consolidation (EWC). The proposed framework does not include an explicit penalty term/Elastic Weight Consolidation (EWC) to preserve the previously learned parameters. Therefore, the proposed method does not enforce theoretical constraints on weight updates but instead relies on knowledge reuse through initialization, followed by standard supervised training on a new dataset. The model is sequentially trained across datasets, where each new training phase begins from previously learned parameters and is optimized using the same loss function and hyperparameters. This method enables efficient knowledge transfer and faster convergence. The incremental weight transfer process is described in Algorithm 1.
**Algorithm 1.** Incremental Weight Transfer**Input:** Current model M, Previous best path P**Output:** Initialized model for training 1If P exists and incremental learning is enabled: 2Load the saved checkpoint from P 3Initialize model M using load state dict(weights, strict = False) 4Continue training M on the new dataset 5Print: “Warm-started from previous best weights” 6Else: 7Initialize model M with random weights 8Print: “Training from scratch” 9End If 10Return M

The proposed model is trained with the hyperparameters given in Table 3.

A pragmatic set of hyperparameters guides the training for the hybrid U-Net with Transformer bottleneck model to ensure stability and performance. The base channel multiplier is 32, meaning the first encoder layer has 32 feature maps, and this number doubles at each downsampling step. Training is sped up with mixed precision via PyTorch’s 2.11.0 GradScaler and stabilized with gradient clipping with a maximum norm of 5.0. We use 4 self-attention layers, each with 8 attention heads, in the Transformer bottleneck to learn global spatial dependencies.

## 4. Results

In this experiment, four publicly accessible datasets and one locally collected dataset are used, including BUS-UCLM, BUSI, TNBC nuclei segmentation, BreastDM, and BCSD-2024. In the proposed BCSD-2024 dataset, the left and right breasts of 10 patients’ mammograms are manually annotated by trained radiologists. The affected regions are then identified by creating a binary mask for each slice. The combined right and left breast slices from 3042 mammography scans of 10 cancer patients whose breasts were chosen at random. Consultant radiologists have already reported bilateral mammography. The suggested dataset BCSD-2024 is used to train a SAM algorithm. To assess the SAM algorithm and determine the dataset’s diagnostic accuracy, 3042 slices from 10 patients were used. Patients aged 36 to 69 participated in the study. The Radiology department at POF Hospital conducted all mammograms. A 256 × 256 × 3-pixel image is included in every patient slice. The images are in JPEG format [29].

The BUS-UCLM dataset includes 683 breast ultrasound images from 38 patients (174 benign, 90 malignant, and 419 normal) taken between 2022 and 2023 using a Siemens ACUSON S2000TM system (Siemens Healthineers, Mountain View, CA, USA). Normal (black), malignant (red), and benign (green) areas are indicated using RGB ground-truth masks [30]. The BUSI-with-GT includes 780 ultrasound images from 600 female patients aged 25–75 years, collected in 2018, and classified into standard, benign, and malignant classes using ground-truth masks. The images are 500 × 500 pixels and in PNG format [31]. The TNBC nuclei outlines dataset includes 40× magnified histopathological H&E-stained images of triple-negative breast cancer tissue, with expertly annotated nuclear masks representing multiple cell types [32]. BreastDM dataset containing 232 cases where 147 are malignant, and 85 are benign, with ground-truth masks for segmentation of breast cancer [33].

The threefold division, training (70%), validation (15%), and testing (15%), was performed for each dataset. The test set was held out for unbiased evaluation; the validation set was used for hyperparameter optimization and monitoring overfitting; and the training set was used to train the model.

Moreover, the data has been split at the patient- or image-level to ensure that no images/patches from the same patient appear in multiple splits. This prevents potential leakage between the training/validation and testing sets.

### 4.1. Evaluation Metrics

**Figure 5:**
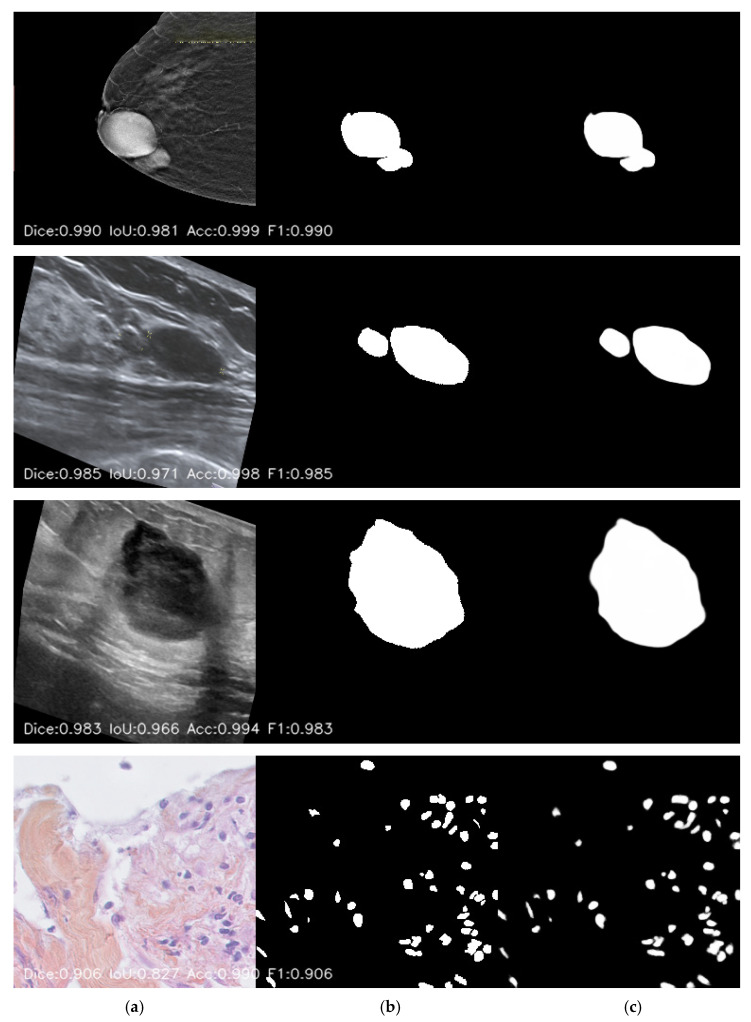
Qualitative segmentation results across different datasets. (**a**) Input images from ultrasound and histopathology modalities, (**b**) corresponding ground-truth masks, and (**c**) predicted segmentation masks generated by the proposed model. The displayed metrics (Dice, IoU, Accuracy, and F1-score) for each example demonstrate the model’s segmentation performance, highlighting strong agreement between the predicted and ground-truth regions.

Model performance is assessed using measures such as the Dice Similarity Coefficient (DSC) and Intersection over Union (IoU), which are commonly used to quantify overlap between predicted and ground-truth labels. All metrics (Dice, IoU, accuracy, AUC) were computed per foreground object and then averaged to mitigate inflation caused by the dominant background class. The segmentation results of the proposed hybrid U-Net model are shown in Figure 5.

In Figure 5, segmentation results are shown for input images from different modalities, including histopathology, ultrasound, and mammography, fed to the proposed model. The performance of the predicted mask is also computed pixel-by-pixel against the ground-truth mask using Dice, IoU, accuracy, and F-score.

### 4.2. Segmentation of Breast Cancer

**Figure 6:**
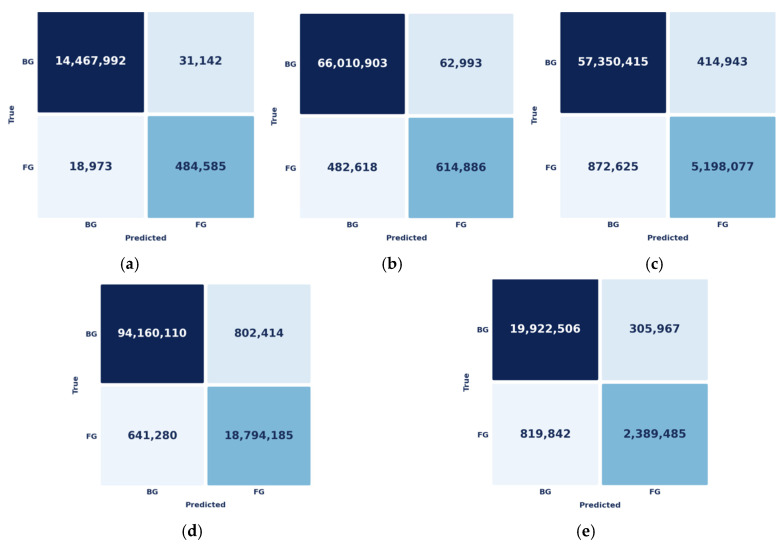
Confusion matrices for the augmentation hybrid model on testing datasets: (**a**) BCSD-2024, (**b**) BUS-UCLM, (**c**) BUSI-with-GT, (**d**) TNBC nuclei, and (**e**) BreastDM. BG: Background, FG: Foreground.

During training, we employ data augmentation across the full setup by horizontally and vertically flipping images, randomly rotating them by 0°, 90°, 180°, or 270°, and scaling them to prevent overfitting. The proposed segmentation model results are presented as confusion matrices for the test samples on benchmark datasets, as shown in Figure 6.

**Figure 7:**
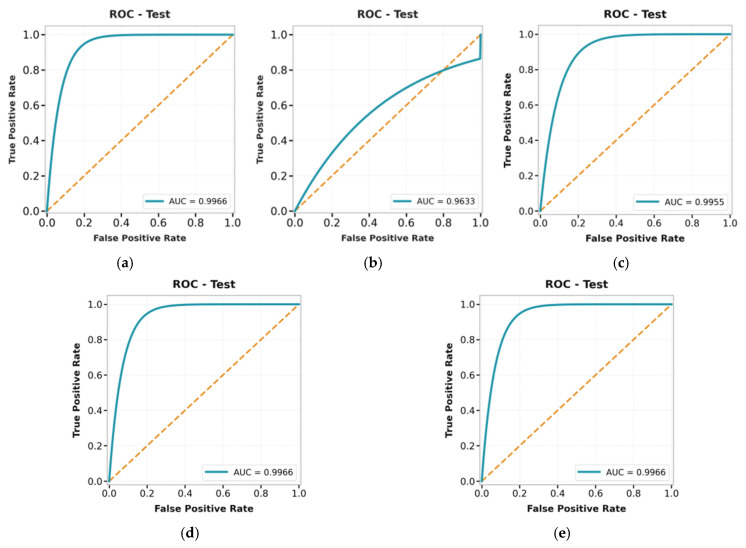
AUC curves for the augmentation hybrid model for the test datasets: (**a**) BCSD-2024, (**b**) BUS-UCLM, (**c**) BUSI-with-GT, (**d**) TNBC nuclei, and (**e**) BreastDM.

In Figure 6, diagonal (background (BG) → BG and foreground (FG) → FG) have the most values, which means that most of the pixels are correctly classified. The off-diagonal values are minimal, indicating few misclassifications between BG and FG. This shows the model distinguishes the classes well despite class imbalance. High Dice scores (>0.97) further confirm strong overlap between the predicted and ground-truth regions. Data augmentation helps by providing the model with more diverse training samples. This lets the model learn different patterns and better detect object boundaries, especially in the foreground. As a result, the proposed model accurately segments breast lesions with fewer errors. The ROC curves indicate that the proposed model clearly distinguishes between the two classes (Figure 7).

In Figure 7, AUC scores are consistently high across most datasets. For example, on the BUS-UCLM dataset, the AUC values remain close across training, validation, and test sets, indicating stable classification performance. The proposed hybrid U-Net model’s performance is evaluated on validation and test image sets, as shown in Table 4, including 95% confidence intervals (CI) for all performance indicators based on bootstrapping with 1000 resamples.

In Table 4, segmentation results are reported for the hybrid U-Net with a Transformer, the U-Net with augmentation, and the U-Net without augmentation. It is noted that all model variants achieved high Dice and IoU scores of approximately 0.98 using BCSD-2024. The hybrid model with binary cross-entropy and without augmentation shows slightly better performance on both the validation and test sets.

On the BUS-UCLM dataset, the hybrid model with augmentation achieves the highest Dice scores among the variants. All variants of the model give better results, close to 0.98–0.99, on BUSI. In this dataset, the hybrid model without augmentation achieves the best test accuracy of 0.99. BreastDM achieved Dice scores of 0.89 to 0.92, which are slightly lower due to complex nuclear structures. The proposed hybrid models achieved excellent results across all breast cancer datasets, with Dice and IoU scores mostly above 0.97. Overall, hybrid-bce-noaug and hybrid-dice-bce-aug are the best-performing models in terms of consistency and accuracy. Although these are challenging datasets, even for the proposed local dataset BCSD-2024, the models still achieve good accuracy and segmentation quality when tested on TNBC nuclei. The hybrid model without augmentation achieves the best segmentation results across most datasets. It achieved the highest scores on test data for the BreastDM, BCSD, BUSI, and BUS-ULCM datasets. The TNBC dataset was the hardest for all model variants. These variants have the lowest Dice scores among others. The results reveal that the proposed hybrid U-Net outperforms baseline models in a statistically consistent manner. Despite the small numerical differences, the confidence intervals show that the improvements are consistent and, in most cases, have little or no overlap with the baseline, indicating statistical significance.

The segmentation report is a more detailed assessment of model performance based on key metrics such as precision, recall, F1-score, and accuracy. These two scores measure how well the model predicted the background and foreground in breast cancer images. The high segmentation consistency is evident from the high accuracies and F1-scores obtained on the test datasets for the proposed model.

On the BCSD-2024 dataset, the proposed model achieves a stable accuracy of ≈0.997 and a recall of ≈0.96. On BUS-UCLM, an accuracy of 0.9918 is achieved in classifying foreground and background pixels. The achieved accuracies were 0.9829 on BUSI, 0.9818 on BreastDM, and 0.9732 on TNBC. This indicates that hybrid models learn global structures well. The proposed model achieves consistent performance across all benchmark datasets and precisely separates pixels into background and foreground. In BCSD-2024, the background score is very high (~0.998), and the foreground also performs well (~0.94–0.97), showing stable tumor detection. In BUS-UCLM, the background remains accurate (~0.992), while the foreground is lower (~0.55–0.78). This drop is due to noise and irregular lesion shapes in ultrasound scans. In the BUSI dataset, background and foreground accuracies are ~0.98–0.99, indicating accurate boundary segmentation. In BreastDM, the model achieves balanced performance on foreground and background, with ~0.96–0.98, indicating stable segmentation results. In TNBC, the background performance is ~0.983, and the foreground region is stable at ~0.87–0.93. The weighted and macro scores remain high across all datasets, indicating balanced learning across each class.

The model provides high accuracy in all benchmark datasets. The background class is detected very well. On the BCSD-2024 dataset, the model performs very well. The weighted average score is also high, indicating a good balance. The model’s performance remains consistent across most datasets. The accuracy remains above 0.97 across all datasets.

In Table 5, recall, precision, and F1-score are measured using one-vs-rest for each class. Therefore, when the background is treated as the positive class, recall denotes the proportion of background pixels correctly identified, while precision denotes the correctness of the predicted background pixels. Due to inherent class imbalance in medical image segmentation, these values might appear counterintuitive but are consistent with the high IoU and Dice scores reported in Table 4. The proposed results model was compared with existing methods, as given in Table 6.

In Table 6, recent work has discussed various convolutional and Transformer-based models for breast cancer segmentation. The segmentation method uses a shared encoder within the multi-task framework to extract rich spatial features. These features are fed to the decoder branch that generates tumor masks by capturing structural and boundary details. The method’s performance is assessed on the BUSI dataset, which achieved a Dice score of 0.752 ± 0.018 [34]. The hybrid DL model uses self-attention layers, U-Net, ResNet34, and multi-scale residual dilated convolution to enhance nuclear segmentation. This model achieves a Dice of 82.31 ± 2.4 on the TNBC nuclei segmentation dataset [35]. The semi-supervised model is applied to segment lesion boundaries using ultrasound imaging, achieving a Dice score of 71.50 ± 1.22 [36]. The self-supervised model is applied to learn the global features from MRI images. Furthermore, peritumoral context restoration is incorporated to permit the model to capture the surrounding tissue dependencies. This model’s performance is evaluated on the BreastDM dataset, which achieved a Dice score of 0.841 ± 0.139 [37].

## 5. Discussion

To address the potential for overfitting due to high IoU and Dice scores, a comprehensive analysis has been conducted across multiple datasets and experimental designs. On the BCSD-2024 dataset, the hybrid BCE with dice loss without augmentation achieves 0.991 Dice score on the validation set, while 0.990 on the test unseen dataset. Similarly, on the BUS-ULCM dataset, the model achieved Dice scores of 0.991 on the validation set and 0.985 on the test set. These smaller differences ensure the model does not overfit to training data. Secondly, across different datasets such as BUS-UCLM, BUSI-with-GT, BreastDM, and TNBC nuclei segmentation, the model maintains stable performance. In particular, the TNBC dataset is more challenging, with test Dice scores of 0.906, further indicating that the model not only memorizes patterns but also adapts to the dataset’s complexity. Thirdly, multiple variants of the model, including a hybrid and a baseline U-Net with and without augmentation, are evaluated, and the results remain consistent across configurations. This consistency across the architecture further validates the robustness of the proposed model. Moreover, data augmentation methods (rotation, flipping, and intensity variation) are applied during training, and the dataset is properly split with no overlap between the training, validation, and test sets, thereby eliminating the possibility of data leakage. The close agreement between validation and test results, combined with consistent performance across independent datasets and model variants, suggests that the proposed model generalizes well without significant overfitting.

## 6. Conclusions

This paper proposes the Incremental Transformer-Enhanced Hybrid U-Net model, trained with an updated storage buffer and an incremental learning framework, which combines a CNN and a Transformer with the U-Net decoder. The model achieves consistent performance across key evaluation metrics, including IoU, Dice coefficient, and accuracy. The main contribution is a new dataset of mammogram images from ten patients at Pakistan Ordnance Factories Hospital. Each image has been manually annotated and validated using ground masks created by skilled radiologists. The proposed dataset consists of medical data, which makes it much more valuable for creating effective comparison breast tumor models than publicly available datasets that are prepared or digital in nature. According to experimental results, the proposed Incremental Transformer-Enhanced Hybrid U-Net model consistently performs well in segmentation on BUS-UCLM and BCSD-2024, achieving nearly 1.0 in accuracy. Conversely, the variability, missing annotations, and diverse imaging conditions in datasets such as BUSI, TNBC Nuclei Segmentation, and BreastDM contributed to a gradual decline in performance across datasets. However, the incremental learning framework demonstrates the model’s ability to retain and adapt to diverse imaging modalities. Focusing on characteristics such as data variety, domain fine-tuning in models, and blending various feature types can further improve system reliability and robustness. This study demonstrates tumor segmentation using corresponding models, which could be an important contribution towards developing better diagnostic equipment in hospitals.

The current framework’s use of basic warm-start initialization in sequential transfer learning is a drawback that can lead to suboptimal generalization and even forgetting previously learned information. To increase stability and maintain performance across several domains, more powerful ongoing learning techniques, such as information distillation, regularization-based approaches, and rehearsal mechanisms, will be investigated in a subsequent study.

## Figures and Tables

**Table 1:** Architecture of the proposed hybrid U-Net model.

Layer	Input Shape	Output Shape	Parameters	Features
Input	(3, 256, 256)	(3, 256, 256)	0	RGB Input Image
Encoder Block 1	(3, 256, 256)	(32, 128, 128)	34,944	Residual Blocks + MaxPool, ReLU
Encoder Block 2	(32, 128, 128)	(64, 64, 64)	279,552	Residual Blocks
Encoder Block 3	(64, 64, 64)	(128, 32, 32)	1,117,184	Residual Blocks
Encoder Block 4	(128, 32, 32)	(256, 16, 16)	4,467,712	Residual Blocks
Bottleneck	(256, 16, 16)	(512, 16, 16)	7,161,856	Double Residual Blocks + ReLU
Transformer	(512, 16, 16)	(512, 16, 16)	33,636,352	4 Layers, 8 Heads, MLP ×4, GELU
Decoder Block 4	(512, 16, 16)	(256, 32, 32)	4,720,128	Skip Connection + Residual Blocks
Decoder Block 3	(256, 32, 32)	(128, 64, 64)	1,180,160	Upsampling + Residual Blocks
Decoder Block 2	(128, 64, 64)	(64, 128, 128)	295,040	Upsampling + Residual Blocks
Decoder Block 1	(64, 128, 128)	(32, 256, 256)	73,792	Upsampling + Residual Blocks
Output Conv	(32, 256, 256)	(1, 256, 256)	33	1 × 1 Convolution

**Table 2:** Parameters of the proposed hybrid U-Net model.

Model	Total Parameters	Parameters of Transformer	Parameters of CNN	Memory
Hybrid U-Net	~52.9 M	33.6 M	19.3 M	~211 MB
Simple U-Net	~9.5 M	0	9.5 M	~38 MB

**Table 3:** Hyperparameters for model training.

Hyperparameters	Values
Base (channels multiplier)	32
Input size	256 × 256
Batch size	4 (adjust to GPU RAM)
Learning rate	1 × 10^−4^
Epochs	Up to 50 with Early Stopping (patience = 12)
Optimizer	Adam
Scheduler	ReduceLROnPlateau (factor = 0.5, patience = 5)
Loss	Dice + BCE
Mixed precision	Enabled if CUDA is available (Grad Scaler)
Gradient clipping	Clip-grad-norm-max-norm = 5.0
Transformer layers	4
Transformer heads	8

**Table 4:** Segmentation results of the proposed hybrid U-Net model.

Datasets	Split	Dice Score (Confidence Interval)	IoU (Confidence Interval)	ACC (Confidence Interval)	F1 (Confidence Interval)
BCSD-2024	BCSD-2024 (hybrid-bce-noaug)				
	Validation	0.991 (0.989–0.993)	0.982 (0.979–0.985)	0.999 (0.998–1.000)	0.991 (0.989–0.993)
	Test	0.990 (0.988–0.992)	0.981 (0.978–0.984)	0.999 (0.998–1.000)	0.990 (0.988–0.992)
	BCSD-2024 (hybrid-dicebce-aug)				
	Validation	0.990 (0.987–0.992)	0.981 (0.977–0.984)	0.999 (0.998–1.000)	0.990 (0.987–0.992)
	Test	0.982 (0.979–0.985)	0.965 (0.961–0.969)	0.998 (0.997–0.999)	0.982 (0.979–0.985)
	BCSD-2024 (unet-dice bce-aug)				
	Validation	0.983 (0.980–0.986)	0.966 (0.962–0.970)	0.998 (0.997–0.999)	0.983 (0.980–0.986)
	Test	0.981 (0.978–0.984)	0.963 (0.959–0.967)	0.997 (0.996–0.998)	0.981 (0.978–0.984)
BUS-UCLM	BUS-UCLM (hybrid-bce-noaug)				
	Validation	0.991 (0.988–0.993)	0.982 (0.978–0.985)	0.999 (0.998–1.000)	0.991 (0.988–0.993)
	Test	0.985 (0.982–0.988)	0.971 (0.967–0.975)	0.998 (0.997–0.999)	0.985 (0.982–0.988)
	BUS-UCLM (hybrid-dicebce-aug)				
	Validation	0.972 (0.968–0.976)	0.945 (0.939–0.951)	0.999 (0.998–1.000)	0.972 (0.968–0.976)
	Test	0.974 (0.970–0.978)	0.950 (0.945–0.955)	0.994 (0.992–0.996)	0.974 (0.970–0.978)
	BUS-UCLM (unet-dicebce-aug)				
	Validation	0.979 (0.975–0.983)	0.959 (0.954–0.964)	0.998 (0.997–0.999)	0.979 (0.975–0.983)
	Test	0.979 (0.975–0.983)	0.960 (0.955–0.965)	0.998 (0.997–0.999)	0.979 (0.975–0.983)
BUSI-with-GT	BUSI-with-GT (hybrid-bce-noaug)				
	Validation	0.978 (0.974–0.982)	0.957 (0.952–0.962)	0.995 (0.993–0.997)	0.978 (0.974–0.982)
	Test	0.986 (0.983–0.989)	0.973 (0.969–0.977)	0.999 (0.998–1.000)	0.986 (0.983–0.989)
	BUSI-with-GT (hybrid-dicebce-aug)				
	Validation	0.982 (0.974–0.982)	0.965 (0.952–0.962)	0.995 (0.993–0.997)	0.982 (0.974–0.982)
	Test	0.975 (0.983–0.989)	0.951 (0.969–0.977)	0.993 (0.998–1.000)	0.975 (0.983–0.989)
	BUSI-with-GT (unet-dicebce-aug)				
	Validation	0.979 (0.978–0.986)	0.959 (0.960–0.970)	0.998 (0.993–0.997)	0.979 (0.978–0.986)
	Test	0.979 (0.971–0.979)	0.960 (0.946–0.956)	0.998 (0.991–0.995)	0.979 (0.971–0.979)
BreastDM	BreastDM (hybrid-bce-noaug)				
	Validation	0.980 (0.976–0.984)	0.960 (0.955–0.965)	0.994 (0.992–0.996)	0.980 (0.976–0.984)
	Test	0.977 (0.973–0.981)	0.955 (0.950–0.960)	0.999 (0.998–1.000)	0.977 (0.973–0.981)
	BreastDM (hybrid-dicebce-aug)				
	Validation	0.972 (0.968–0.976)	0.946 (0.941–0.951)	0.988 (0.985–0.991)	0.972 (0.968–0.976)
	Test	0.971 (0.967–0.975)	0.944 (0.939–0.949)	0.991 (0.989–0.993)	0.971 (0.967–0.975)
	BreastDM (unet-dicebce-aug)				
	Validation	0.981 (0.977–0.985)	0.963 (0.958–0.968)	0.989 (0.987–0.991)	0.981 (0.977–0.985)
	Test	0.976 (0.972–0.980)	0.953 (0.948–0.958)	0.992 (0.990–0.994)	0.976 (0.972–0.980)
TNBC nuclei segmentation	TNBC (hybrid-bce-noaug)				
	Validation	0.928 (0.920–0.936)	0.866 (0.855–0.877)	0.987 (0.984–0.990)	0.928 (0.920–0.936)
	Test	0.906 (0.898–0.914)	0.827 (0.815–0.839)	0.990 (0.987–0.993)	0.906 (0.898–0.914)
	TNBC (hybrid-dicebce-aug)				
	Validation	0.909 (0.900–0.918)	0.883 (0.872–0.894)	0.978 (0.974–0.982)	0.909 (0.900–0.918)
	Test	0.904 (0.895–0.913)	0.826 (0.814–0.838)	0.977 (0.973–0.981)	0.904 (0.895–0.913)
	TNBC (unet-dicebce-aug)				
	Validation	0.919 (0.910–0.928)	0.850 (0.838–0.862)	0.982 (0.978–0.986)	0.919 (0.910–0.928)
	Test	0.897 (0.888–0.906)	0.814 (0.802–0.826)	0.986 (0.982–0.990)	0.897 (0.888–0.906)

**Table 5:** Pixel-wise comparison between the segmented lesion (foreground) and surrounding tissue (background).

Datasets	Background (BG)	Foreground (FG)	Accuracy	Macro Avg	WeightedAvg	Precision	Recall	F1
BCSD-2024	☑					0.9983	0.9978	0.9981
		☑				0.9395	0.9529	0.9462
			☑					0.9963
				☑		0.9689	0.9754	0.9721
					☑	0.9964	0.9963	0.9963
BUS-UCLM	☑					0.9927	0.9990	0.9959
		☑				0.9071	0.5587	0.6915
			☑					0.9918
				☑		0.9499	0.7789	0.8437
					☑	0.9913	0.9918	0.9909
BUSI-with-GT	☑					0.9884	0.9928	0.9906
		☑				0.9261	0.8855	0.9053
			☑					0.9829
				☑		0.9572	0.9392	0.9480
					☑	0.9827	0.9829	0.9827
BreastDM	☑					0.9895	0.9886	0.9890
		☑				0.9447	0.9488	0.9467
			☑					0.9818
				☑		0.9671	0.9687	0.9679
					☑	0.9818	0.9818	0.9818
TNBC	☑					0.9836	0.9865	0.9851
		☑				0.8796	0.8571	0.8682
			☑					0.9732
				☑		0.9316	0.9218	0.9267
					☑	0.9729	0.9732	0.9730

**Table 6:** Results of the proposed segmentation model compared to the existing methods.

Ref#	Year	Dataset(s)	Dice
[34]	2025	BUSI	0.752 ± 0.018
[35]	2026	TNBC	82.31 ± 2.4
[36]	2026	BUS-UCLM	71.50 ± 1.22
[37]	2025	BreastDM	0.841 ± 0.139
Proposed Methodology		Proposed BCSD-2024	0.982
		BUS-UCLM	0.974
		BUSI	0.975
		TNBC nuclei segmentation	0.904
		BreastDM	0.971

## Data Availability

The original contributions presented in this study are included in the article. Further inquiries can be directed to the corresponding author.
